# Male and Female Fertility: Prevention and Monitoring Hodgkin’ Lymphoma and Diffuse Large B-Cell Lymphoma Adult Survivors. A Systematic Review by the Fondazione Italiana Linfomi

**DOI:** 10.3390/cancers13122881

**Published:** 2021-06-09

**Authors:** Simonetta Viviani, Valentina Caccavari, Chiara Gerardi, Safaa Ramadan, Eleonora Allocati, Carla Minoia, Attilio Guarini, Anna Di Russo

**Affiliations:** 1 Division of Onco-Hematology IEO, European Institute of Oncology IRCCS, 20141 Milan, Italy; safaa.ramadan@ieo.it; 2 Assisted Reproduction Unit, Istituto Clinico Città Studi, 20131 Milan, Italy; valentina.caccavari@ic-cittastudi.it; 3 Istituto di Ricerche Farmacologiche “Mario Negri” IRCCS, 20156 Milan, Italy; chiara.gerardi@marionegri.it (C.G.); eleonora.allocati@marionegri.it (E.A.); 4 Department of Medical Oncology, National Cancer Institute, Cairo University, 11796 Cairo, Egypt; 5 Hematology Unit—IRCCS Istituto Tumori “Giovanni Paolo II”, 70124 Bari, Italy; carlaminoia@libero.it (C.M.); attilioguarini@oncologico.bari.it (A.G.); 6 Department of Radiation Oncology, Fondazione IRCCS Istituto Nazionale dei Tumori di Milano, 20133 Milan, Italy; anna.dirusso@istitutotumori.mi.it

**Keywords:** hodgkin lymphoma, diffuse large B-cell lymphoma, chemotherapy, radiation therapy, fertility preservation, infertility, gonadotoxicity, oocyte cryopreservation, ovarian tissue cryopreservation, pregnancy

## Abstract

**Simple Summary:**

Patients with Hodgkin lymphoma or diffuse large B-cell lymphoma treated with chemotherapy, with or without radiation therapy on pelvic nodes, may suffer permanent damage to their reproductive function, which significantly affects their quality of life. Established gamete cryopreservation techniques, including the freezing of embryos, oocytes, spermatozoa, ovarian and testicular tissue, can, nowadays, be offered to any adult patient candidate receiving gonadotoxic therapy who is interested in preserving future fertility. In order to offer updated information on anticancer treatment harm and to better advise patients on fertility preservation options, Fondazione Italiana Linfomi (FIL) researchers conducted this systematic review to evaluate the frequency of treatment-related infertility, fertility preservation options, fertility assessment measures, and the optimal interval between end of treatment and conception in adult lymphoma patients.

**Abstract:**

Background: Adult patients with Hodgkin lymphoma (HL) and diffuse large B-cell lymphoma (DLBCL) have prolonged survival but face the risk of treatment-induced impaired fertility. This systematic review, conducted by Fondazione Italiana Linfomi (FIL) researchers, aims to evaluate the incidence of treatment-related infertility, fertility preservation options, fertility assessment measures, and the optimal interval between the end of treatment and conception. Methods: MEDLINE, the Cochrane Library, and EMBASE were systematically searched up to September 2020 for published cohort, case–control, and cross-sectional studies on fertility issues. Results: Forty-five eligible studies were identified. Gonadotoxicity was related to sex, type and dosage of treatment, and, in females, to age. After receiving alkylating-agent-containing regimens, less than 30% of males recovered spermatogenesis, and 45% of females ≥30 years in age retained regular menstrual cycles. Sperm cryopreservation was offered to the majority of patients; sperm utilization resulted in a 33–61% pregnancy rate. After ovarian tissue transplantation, the spontaneous pregnancy and live birth rates were 38% and 23%; after IVF, the live birth rate was 38.4%. No data could be extracted on the utilization rate of cryopreserved mature oocytes. The results of studies on GnRH analogs are controversial; therefore, their use should not be considered an alternative to established cryopreservation techniques. Sperm count, FSH, and inhibin-B levels were appropriate measures to investigate male fertility; serum AMH levels and antral follicle count were the most appropriate markers for ovarian reserve. No data could be found regarding the optimal interval between the end of treatment and conception. Conclusions: The risk of infertility should be discussed with adult lymphoma patients at the time of diagnosis, and fertility preservation options should be proposed before first-line treatment with alkylating-agent-containing regimens.

## 1. Introduction

The majority of adult patients diagnosed with Hodgkin lymphoma (HL) and non-Hodgkin (NHL) diffuse large B-cell lymphoma (DLBCL) have favorable life expectancy with current treatment strategies, which have resulted in improvements in long-term outcomes [1,2,3]. Unfortunately, most of these treatment options adversely affect fertility. In fact, in recent years, infertility has become a relevant complication of cancer treatment to be discussed at the time of diagnosis, particularly for patients of childbearing age [4]. The majority of lymphoma treatment protocols in adult patients consist of combination chemotherapy (CT) regimens containing alkylating agents (e.g., cyclophosphamide, chlorambucil, mechlorethamine, procarbazine, ifosfamide, cisplatin or oxaliplatin, carboplatin, melphalan, dacarbazine) that are known to cause depletion of the germinal epithelium and aplasia of germinal cells in males and oocyte destruction and follicular depletion in females, with deleterious effects on both spermatogenesis [5,6] and ovarian reserve [7]. Nonalkylating agents such as anthracyclines, bleomycin, etoposide, mitotic spindle inhibitors (such as the vinca derivatives vincristine, vinblastine, or vinorelbine), gemcitabine, steroids, and rituximab are less frequently associated with gonadal dysfunction. Gonadotoxicity of recently introduced new drugs in the therapeutic armamentarium of HL and NHL, such as brentuximab vedotin, checkpoint inhibitors, polatuzumab, ibrutinib, acalubrutinib, and venetoclax, is still unknown. The risk of infertility also depends on the patient’s age at treatment, type and stage of the disease, and type and dosage of the CT regimens [7,8,9]. In addition, radiotherapy (RT) to the pelvis, given alone or in combination with CT, or high-dose chemotherapy (HD-CT) with autologous stem cell support (ASCT) and/or with allogeneic bone marrow transplant (allo-SCT) in the course of the disease may lead to severe injury to the ovarian reserve or testicular germ cells and infertility. Since having biological children is an important aspect of quality of life after cancer, fertility preservation has become an integral part of the management of patients with hematological malignancies. Therefore, over the last two decades, many efforts have been made to improve the cryopreservation of semen, oocytes, ovarian tissue, and embryos in these patients [10].

The main objective of this study is to systematically review the literature on fertility complications in adult HL and DLBCL survivors following frontline CT combinations, with or without RT, or following salvage HD-CT with ASCT or allo-SCT. Specifically, Fondazione Italiana Linfomi (FIL) researchers evaluated, in this work, the incidence of long-term treatment-related infertility, the available fertility preservation options, the most appropriate fertility assessment examinations in the follow-up period, and the optimal time interval from end of treatment to future reproduction. This analysis aims at providing practitioners with more accurate data on the gonadal toxicity of commonly used treatment regimens and fertility preservation options. To do this, our research was focused on answering the following five questions:

1. Which adult female and male HL or DLBC patients are eligible for fertility preservation procedures?
2. What are the fertility preservation procedures available to adult male and female HL or DLBCL patients?
3. Is the use of gonadotropin-releasing hormone analogs (GnRHa) during treatment effective in reducing the risk of infertility in adult premenopausal female HL or DLBCL patients receiving CT regimens containing or not containing alkylating agents, HD-CT, or RT to the pelvic nodes?
4. What are the most appropriate fertility investigations in adult male and female HL or DLBCL patients treated with CT and/or pelvic irradiation?
5. What is the best interval to recommend before conceiving after the end of treatment?

Full details on PICOs questions are reported in Table 1.

## 2. Materials and Methods

This systematic review is part of a series of analyses in a research program exploring long-term complications in adult lymphoma survivors and their management. It also intends to provide support to FIL position statements regarding this subject. The scope of the position statements and the clinical queries and Population/Intervention/Control/Outcome studies (PICOs) for each question were discussed and agreed on by the FIL Long-Term Survivor Committee and presented at the FIL congress in 2019. We used the Preferred Reporting Items for Systematic reviews and Meta-analyses (PRISMA) guidelines to report the results [11].

### 2.1. Study Identification

MEDLINE (via PubMed), the Cochrane Library, and EMBASE were systematically searched from 1 January 1990 to 30 September 2020 with no language or publication-type restrictions. Search terms included extensive controlled vocabulary (MeSH and EMTREE) and free-text keywords, combining the conditions (classical Hodgkin disease, Diffuse Large B-cell Lymphoma), interventions (e.g., chemotherapy and radiotherapy), and outcomes of interest (e.g., gonadotoxicity, infertility, fertility, premature ovarian insufficiency or failure, azoospermia, spontaneous reproduction, GnRH analogs, oocyte cryopreservation, ovarian tissue cryopreservation, and semen cryopreservation). Details on the search strategies can be found in Table S1. We checked the reference lists of relevant works to further retrieve studies and congress abstracts and searched study registries for unpublished or ongoing studies.

### 2.2. Eligibility Criteria

We included both primary studies (randomized controlled trials, prospective and retrospective cohort studies, and registry studies) and systematic reviews, including these study designs. We included studies involving adult (aged ≥18 years at diagnosis) male and female HL or DLBCL patients in complete remission for at least 12 months after first-line or second-line treatment, including ASCT or allo-SCT. These studies assessed the incidence of azoospermia or premature ovarian failure (POF) after treatment, the percentage of cryopreserved gametes or ovarian tissue utilization, the number of spontaneous conceptions, assisted reproductive technique (ART) pregnancies, the number of live births, the efficacy of GnRHa, the effect of therapy on biochemical indicators of ovarian or testicular function, the time to conception, and spontaneous pregnancies after ≥12 months from the end of anticancer treatment.

### 2.3. Risk of Bias and Quality of Evidence Assessment

We revised the methodological quality of the included systematic reviews using the AMSTAR 2 tool [12], the risk of bias for the RCTs using the Cochrane Risk of Bias (ROB) [13], and the quality of cohort and registry studies using the New Castle Ottawa Scale (NOS) [14]. The risk of bias and quality of evidence assessments were done by one reviewer and checked by another and are reported in Table S2.

### 2.4. Study Selection and Data Extraction

Three reviewers (S. Viviani, V. Caccavari, and A. Di Russo) independently screened the titles and abstracts to select the studies for each PICO. One reviewer reviewed the full-text publication to confirm its eligibility and extracted the relevant information from the included trials. A second reviewer checked the eligibility and data extraction to increase the accuracy of the process. Any discrepancies were resolved by consensus and arbitration by a third author.

Data collected from each study comprised the following predefined items: (1) study identifier (first author, year of publication); (2) reference; (3) other publication; (4) study design; (5) population; (6) study duration; (7) follow-up; (8) sample size; (9) intervention/control group (10) outcome measure; (11) main results; (12) conclusion; (13) risk of bias/quality assessment. A predefined spreadsheet (Excel 2007, Microsoft Corporation^®^) was used for data extraction and is reported in Table S3.

### 2.5. Data Synthesis

Due to the expected heterogeneity in the searched studies in terms of methodology, design, and the planned endpoints and outcomes to analyze as well as the study population, we did not plan for a meta-analysis. In addition, not all studies included were randomized controlled trials; some were observational. For each clinical question, the studies providing relevant information were summarized narratively and tabulated to highlight the similarities and differences in methods and results.

## 3. Results

### 3.1. PICO 1: Which Female and Male HL or DLBC Patients Are Eligible for Fertility Preservation Procedures?

**Figure 1:**
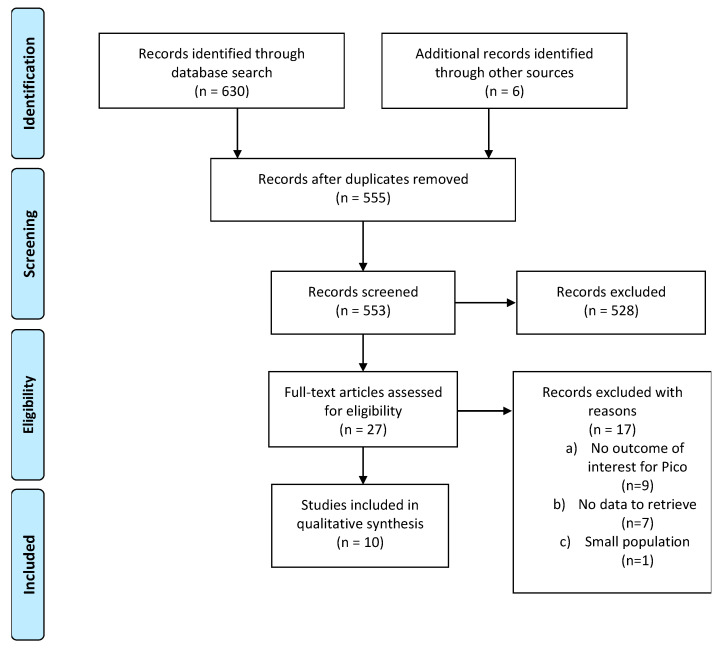
Prisma flow-chart on female HL or DLBCL patients eligible for fertility preservation procedures.

The initial search produced 634 potentially relevant articles. After removing duplicates, 553 abstracts were assessed for eligibility, and 25 full texts were considered for subsequent assessment. Eight studies were included in the final analysis after full-text evaluation. Details of the whole screening process, including the reasons for full-text exclusion, are reported in Figure 1 (PRISMA flow-chart).

Of the eight included studies, two were retrospective studies, four were prospective observational studies, and two were systematic reviews. Details on the eligibility of the studies and data extraction are reported in Table S3. The patient population of these studies received treatment between 1985 and 2019. The majority of publications focused on HL patients, while studies reporting fertility evaluation in NHL patients did not specify the rate of DLBCL.

### 3.2. PICO 1a. Which Female HL or DLBC Patients Are Eligible to Fertility Preservation Procedures?

To answer this question, the studies that have identified females at the highest risk of infertility after cancer treatment were used in the analysis. In females, the incidence of infertility was evaluated by measurement of FSH (follicle-stimulating hormone), LH (luteinizing hormone), and AMH (anti-Müllerian hormone) levels and by data on menstrual status, the occurrence of amenorrhea, menopausal status, and the occurrence of pregnancy, mainly through questionnaires.

Behringer et al. evaluated gonadal function in patients enrolled in HD 13–15 trials for at least one year after the end of treatment for HL [15]. Menstrual cycle recovery was reported in more than 90% of female patients treated with ABVD (doxorubicin, bleomycin, vinblastine, dacarbazine), whereas menstrual recovery after escalated-BEACOPP (eBEACOPP; bleomycin, etoposide, doxorubicin, cyclophosphamide, vincristine, procarbazine, prednisone), an alkylating-containing regimen, was related to age at the start of treatment. In specific, 82% of females younger than 30 years regained a regular menstrual cycle compared to 45% in the older age group (*p* < 0.001) [16].

Comparing the gonadotoxic effects of ABVD and eBEACOPP, post-treatment serum AMH was 2.2 vs. 0.1 µg/L at age 18–29 years and 0.7 vs. 0.0 µg/L at age 30–45 years, indicative of a severe reduction in ovarian reserve after eBEACOPP [15]. We judged this study by NOS to be, in general, of good quality, in particular with regard to selection, number of cases evaluated, and adequacy of follow-up. Full assessments of risk of bias are reported in the supplementary material (Table S3).

In another study, Falorio S. et al. conducted a retrospective multicenter study to evaluate the prevalence and risk factors for impaired gonadal function in female HL survivors [17]. The median age at diagnosis was 25 years, and with a median follow-up of 7 years, 25% of patients developed gonadal dysfunction. In a multivariate analysis, age at diagnosis >30 years (*p* = 0.005), alkylating agents (*p* = 0.03), number of treatments (*p* < 0.001), and no use during treatment of oral contraceptives (*p* = 0.005) or GnRHa (*p* < 0.001) were associated with reduced gonadal function. The quality of this study was considered intermediate, with the main bias represented by the absence of selection criteria of the examined population. 

Anderson et al. retrospectively estimated the effect of two ABVD cycles followed by four AVD (doxorubicin, vinblastine, dacarbazine) cycles versus two ABVD cycles followed by four eBEACOPP cycles on ovarian function, evaluating the levels of reproductive hormones [18]. FSH and LH levels increased during treatment in both groups, then decreased again to basal levels in the ABVD/AVD group, whereas in the eBEACOPP group, serum levels did not decline again (*p* > 0.0001). In the ABVD/AVD (doxorubicin, vinblastine, dacarbazine) group, AMH decreased from a median of 9.8 pmol/L before treatment to 1.7 pmol/L at the end of treatment (*p* < 0.0001). Subsequently, one year after the end of CT, AMH concentrations increased to 10.5 pmol/L. In the BEACOPP group, the concentration of AMH decreased from a median of 6.8 pmol/L to 0.08 pmol/L at the end of treatment (*p* < 0.0001). After treatment, however, AMH concentrations showed very little recovery (median 0.11 pmol/L at 1 year), and patients aged over 35 years were more likely to experience premature ovarian failure (POF). The quality (NOS scale) of this study was high: selection and exposure were well defined.

The risk of infertility has not been extensively studied after autologous hematopoietic cell transplantation (ASCT), which represents the salvage treatment of choice in refractory or relapsed young patients with HL or DLBCL. Blumenfeld et al. analyzed the rate of POF in HL and NHL patients receiving BEAC (BCNU/carmustine, etoposide, cytarabine, cyclophosphamide) or BEAM (BCNU/carmustine, etoposide, cytarabine, melphalan) as conditioning regimes prior to ASCT, reporting a POF rate of 82% [19]. The quality of this study was globally good: the representativeness, selection, and exposure were good as well as the measurements of outcomes.

### 3.3. PICO 1b. Which Male HL or DLBC Patients Are Eligible for Fertility Preservation Procedures?

Male infertility after lymphoma treatment was evaluated by semen analysis, hormone levels (mainly of FSH and inhibin B), and successful fatherhood.

It should be noted that the pretreatment semen quality of adult males with HL and NHL is significantly lower compared to healthy controls. The mechanism of this dysfunction is not known [20,21,22,23,24].

For example, Botchan et al. compared sperm quality in 65 HL and 18 NHL patients, referred prior to treatment for sperm preservation, to 190 healthy volunteers. There was a statistically significant difference in sperm concentration (10^6^/mL) (75 ± 4.4 vs. 136.8 ± 5.5; *p* < 0.01) and motility (%) (37.6 ± 0.9 vs. 47.4 ± 0.8; *p* < 0.01) [20]. In addition, NHL patients had better sperm variables than HL patients in terms of sperm mobility (43.5% vs. 36.0%; *p* = 0.0001), total motile sperm count (105 vs. 88 × 10^6^/mL; *p* = 0.021), normal morphology (12.0% vs. 9.9%; *p* = 0.01), and post-thaw forward motility concentration (8.7 vs. 8.1 × 10^6^/mL; *p* = 0.017). The quality of this study was evaluated with the NOS case–control scale, and it was high. Study population and control were well balanced, as well as exposure and outcome.

Di Bisceglie et al. also serially assessed various semen parameters and assessed hormonal levels (FSH, LH, inhibin B) at the time of semen cryopreservation and then at 6, 12, 18, 24, and 36 months after treatment in 125 HL and 94 NHL patients [21]. HL patients had been treated with four to six cycles of ABVD, and normospermia was documented in 36%, 64%, 80%, and 83% at 12, 18, 24, and 36 months, respectively. FSH levels were higher at 6, 12, 18 and 24 months (*p* < 0.01). Inhibin B levels showed a decline at 6 months (*p* < 0.001), with no other statistically significant variations (compared to baseline values) at the other time points. NHL patients were treated with three to six cycles of R-CHOP (rituximab–cyclophosphamide, doxorubicin, vincristine, prednisone) and RT (30–36 Gy). Normospermia was detected in 45%, 73%, 77%, and 78% of patients at 12, 18, 24, and 36 months, respectively. FSH increased from 6 months onwards (*p* < 0.05) and returned to baseline levels at 18 and 36 months. On the other hand, inhibin B decreased at 6 months (*p* < 0.01), with a return to baseline levels at 18 months. Defective spermatogenesis was still evident at 6 and 12 months, while an improvement of seminal parameters was observed after 18 months after the end of treatment. An overall increase in sperm concentration was recorded about 18 months from treatment completion. However, it was not possible to identify the factors predicting the evolution of every single case “a priori”. Therefore, the authors advised semen cryopreservation in similar types of patients. The quality of this study was intermediate (NOS scale): the case definition was good, as well as the follow-up period.

Harel et al. also confirmed a high percentage, up to 80%, of semen abnormality in HL patients before treatment regardless of their age. The type of chemotherapy regimen was a risk factor for infertility, with significant association with the incorporation of alkylating agents [22]. After ABVD, the rate of azoospermia ranged from 0% to 8%; after ABVD and radiotherapy on pelvic nodes, azoospermia was reported in 2% to 11% of patients, whereas after eBEACOPP, abnormal sperm or an increase in serum FSH levels were observed in 90% to 100% of cases. Consequently, the authors recommended semen cryopreservation before first-line treatment with alkylating agents (such as BEACOPP or salvage therapy for relapse) and considered the procedure optional in cases of localized HL treated with ABVD and radiotherapy.

In a study by Bujan L et al., sperm features such as semen quality, sperm DNA, and chromatin assessments were assessed pre- and postchemotherapy in 75 HL and NHL patients compared to 257 fertile men as control [23]. No risk factor could be detected in patients who already presented defective sperm qualities. Sperm count, total sperm count, motility, and vitality were reduced markedly at T3 and T6 months from the end of treatment. Recovery of the mean sperm count occurred at T12 only in patients treated with ABVD with or without radiotherapy. At 24 months, 7% of patients still had azoospermia, and, at 48 months, >90% of patients regained a normal sperm count after ABVD, with or without radiotherapy, vs. 61% after CHOP chemotherapies. In multivariate analyses, only pretreatment total sperm count was related to recovery. With regard to the control fertile men, at the pretreatment time point, lymphoma patients showed more alterations in sperm chromatin and DNA fragmentation, and this difference remained over T3 and T6 months of follow-up despite improvements. The quality of this study was, in general, good: selection of cases, measurement of outcome, and duration of follow-up were adequate.

Paoli D et al. also retrospectively examined semen parameters in terms of sperm concentration, total sperm number, progressive motility, and morphology in 519 HL patients before therapy [24]. Of these, 75% were normozoospermic prior to treatment, and 15 patients already had azoospermia. In 202 ABVD-treated patients, a serial longitudinal analysis of semen quality could be continued at 6 (T6), 12 (T12), and 24 (T24) months after treatment completion. In contrast, 42 patients who were pre- and post-BEACOPP, COPP/ABVD, OPP/ABVD, or MOPP and had inguinal radiotherapy were evaluated at variable observation times (from T0 to 16 years after treatment). Investigators of this study found that sperm concentration and total sperm number dropped markedly at T6 and T12 (*p* < 0.001 and *p* < 0.01, respectively) after ABVD. At T6, progressive motility (*p* < 0.001) and the frequency of abnormal sperm forms were significantly different from baseline T0 (*p* < 0.01). Importantly, at T24, sperm quality, sperm concentration, total sperm number, and abnormal forms returned to pretherapy values. Unfortunately, and as reported in other studies, six cycles or more of BEACOPP, COPP/ABVD, OPP/ABVD, or MOPP resulted in permanent azoospermia. Even with less than six cycles, spermatogenesis remained compromised for 3–5 years, while semen quality remained greatly weakened. The quality (NOS scale) of this study was good: selection and exposure were well defined; however, the follow-up periods were heterogeneous, and the number of patients treated with chemotherapy regimens other than ABVD was low; thus, no definitive conclusions could be drawn on the recovery of spermatogenesis after BEACOPP.

Poorvu’s review indicated that conditioning chemotherapy and radiation frequently led to severe impairment of male fertility [25]. In fact, recovered spermatogenesis and normal testicular function were described in <30% of men following ASCT, which was even lower after more significant gonadotoxic exposure. The quality of these two studies (AMSTAR) was good, although the literature search strategy and the inclusion/exclusion criteria were not well established. The definition of criteria used to define the risk of bias of the studies included in the review was missing.

### 3.4. PICO 2. What Are the Fertility Preservation Procedures Available to HL or DLBCL Male and Female Patients?

**Figure 2:**
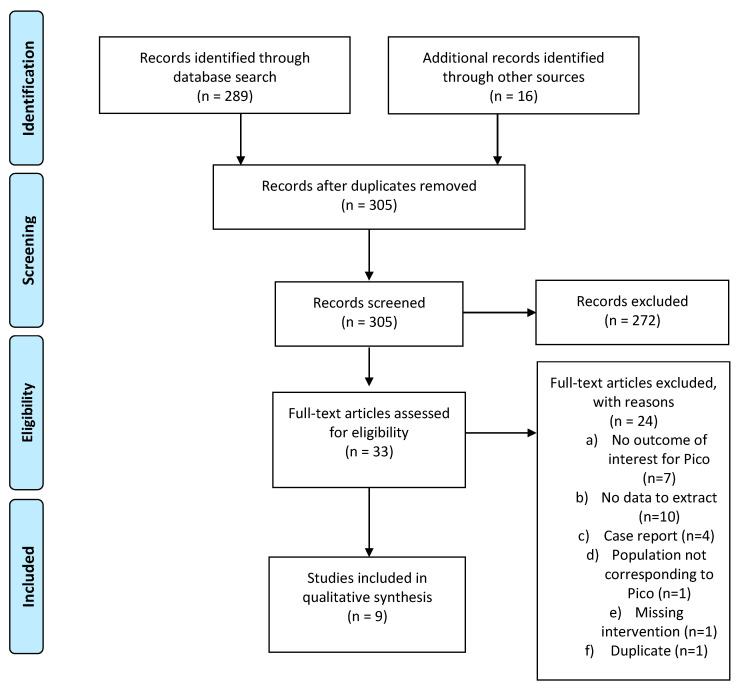
PRISMA flow-chart on fertility preservation procedures available to HL and DLBCL patients.

Thirty-three relevant publications were retrieved as full text out of 305 publications screened. Of these, 21 were excluded and 9 studies were included in the final sample of analysis: 3 studies regarding PICO 2a, and 6 studies regarding PICO 2b. Details of the whole screening process, including the reasons for full-text exclusion, are reported in Figure 2 (PRISMA flow-chart PICO 2), and the details of eligibility and data extraction are reported in Table S3.

### 3.5. PICO 2a. What Are the Fertility Preservation Procedures Available to HL or DLBCL Male Patients?

Blackhall F.H. et al. retrospectively analyzed a cohort of 115 HL patients selected by crossmatching the names of all newly diagnosed male patients registered at Christie Hospital with the names of patients referred, in the same period of time, for semen cryopreservation from the reproductive medical database of St Mary’s Hospital, Manchester, between January 1978 and December 1990 [26]. Patients had a median age of 24 years and had received MVPP (mechlorethamine, vinblastine, procarbazine, prednisone) or a ChlVPP/EVA hybrid (chlorambucil, vinblastine, procarbazine/etoposide, vincristine, doxorubicin) CT, either as an adjuvant treatment following RT in early-stage disease or for advanced-stage disease, followed by RT to sites of initial bulk or residual masses, regimens considered to have similar gonadal toxicity [27]; 74 out of 115 requested a semen analysis after treatment at least once. After a median post-treatment follow-up of 2.9 years, azoospermia was seen in 91.8% of patients and severe oligo-azoospermia in 5.4%. After a median follow-up of 5 years, 83.5% of patients had azoospermia, 13 patients (17.5%) recovered a degree of spermatogenesis, and 4 of them (5.4%) fathered spontaneously. Among the 113 patients with available data, the rate of frozen sperm utilization at 10 years from storage was 27%. Thirty-three couples received assisted reproductive techniques (ART), most of which consisted of artificial insemination with homologous sperm (AIH) (n = 28) since in vitro fertilization (IVF) was offered after 1984 and intracytoplasmic sperm injection (ICSI) from 1995; intrauterine insemination (IUI) replaced AIH in 1995; 10 pregnancies were reported and 11 live births by 8 couples were described, with a 24.2% success rate in couples attempting ART. The population examined was representative of the average population of patients cryopreserving sperm; only two patients were lost to follow-up, and a follow-up length of 10 years is acceptable in a population with an average age of 24 years at cryopreservation. For these reasons, the risk of bias of the study was considered low, although no information was given about men who did not cryopreserve.

In a study by Depalo et al., the data of various cancer patients referred for pretreatment semen cryopreservation for seminoma or other testicular tumors and HL between 1999 and 2015 were retrospectively explored [28]. A total of 173 HL patients (mean age 25.8 years) with available information revealed that the utilization rate of cryopreserved semen was 3.4% (6/173). The reported ART pregnancy rate was 33.3%, but no records on live births were available nor the follow-up length.

Van der Kaaij et al. reported the results of a large representative sample of HL survivors with a long-term follow-up [29]. Investigators in 2008 distributed a Life Situation Questionnaire (LSQ) to male patients included in the H2-H9 EORTC-GELA trials [30] who were alive at the last follow-up. Among the 1849 survivors approached, 902 returned a completed LSQ and were eligible for analysis. The median age at treatment start was 31 years; the median follow-up was 13 years (range, 5–36). Among responders, 363/902 (40%) had their semen cryopreserved before starting treatment, and, of them, 78 (21%) used the semen. With the use of ART in 78 patients who conceived with cryopreserved sperm, 48 pregnancies were recorded in 128 survivors who did not father spontaneously (37.5%). Thus, the use of cryopreserved semen resulted in a pregnancy rate of 61.5% (48/78). The main reasons given by patients who did not use the frozen semen were: fathered spontaneously (45%) or had not yet tried to father children (47%) or other reasons such as insufficient semen quality or other not-specified reasons. The spontaneous conception rate was 23% (209/902) among all responding survivors and 62% (209/334) among those with a wish for children. These data confirm that sperm cryopreservation at diagnosis is the most valuable method of preserving male reproductive potential and can be widely offered to patients. The study was based on a large representative population of exposed patients, with a long follow-up period. The main bias could come from the self-reported outcome.

We did not find relevant abstracts or data in the selected full-text articles on the cryopreservation of testicular tissue, which is still considered an experimental procedure.

### 3.6. PICO 2b. What Are the Fertility Preservation Procedures Available to HL or DLBCL Pre-Menopausal Female Patients?

Ovarian tissue cryopreservation (OTC) is the most promising fertility preservation procedure that is also available to prepubertal girls; it is increasingly being offered to patients before and even after gonadotoxic treatment. Data on its safety and efficacy are growing, but the proportion of patients who actually succeed in freezing their ovaries is not known.

Rosendahl et al. retrospectively examined data on the ovarian function of 92 women who underwent unilateral ovariectomy for OTC before chemotherapy between 1999 and 2007 [31]. Twenty-six of them had a diagnosis of lymphoma (23 HL, 3 NHL). In the whole cohort, the mean age at diagnosis and at the last follow-up were 25.4 (range, 9–37) and 28.7 years (range, 18–41), respectively. Eleven HL patients received ABVD, one received ABV (doxorubicin, bleomycin, vinblastine); 11 had one or more of the following regimens, either as first-line or salvage treatment: BEACOPP, COPP (cyclophosphamide, vincristine, procarbazine, prednisone), BEAM, MIME (mitoguazone, ifosfamide, methotrexate, etoposide); 4 patients received an ASCT, 18 received RT to supradiaphragmatic nodes and 1 received TBI. After a mean follow-up of 34 months (range, 18–75) from cryopreservation, 40% of patients experienced amenorrhea or had irregular menstrual cycles. Hence, although 60% of HL patients regained a regular monthly menstrual cycle, only 11% could return to normal cycles after ASCT.

The degree of ovarian damage is largely influenced by the type of CT regimen received. In this context, patients treated with COPP, BEACOPP, MIME, or BEAM, compared to ABVD, had a lower antral follicle count, a lower level of AMH, and significantly higher FSH levels of >15; 6 of 26 lymphoma patients (23%) conceived, with 3 of them using ovarian tissue transplantation (OTT). A total of 4 live births or ongoing pregnancies were registered (17.3%), 3 without (3/26 = 11.5%) and 1 after OTT (3.8%). In this analyzed cohort, the follow-up length was considered adequate, and data were mainly based on medical records; the major limitation of the study was represented by the small sample size. 

The outcome of OTT was addressed in two prospective cohort studies. In one of them by Meirow et al., OTC was performed by laparoscopic removal of one-half or two-thirds of one ovarian cortex, either before or after CT, in 20 cancer patients, including 8 out of 13 HL or NHL patients who had received CT prior to ovarian tissue harvesting [32]. Patients requested OTT, on average, at 5.6 years after OTC. Before OTT, 12 out of 13 patients had no menses for one year and hormone profiles consistent with a menopausal status; 1 patient showed some ovarian activity but was infertile. Various methods were applied to rule out possible infiltration by cancer cells. Thawing was followed by a mini-laparotomy for auto-transplantation, with insertion of the tissue in subcortical tunnels or beneath the peritoneum in the broad ovarian ligament when there was insufficient space within the atrophic ovaries. Ovarian function with documentation of menstrual bleeding, repeated measurements of FSH and E2 (estradiol-2), and sonographic evaluations were closely monitored. After a median follow-up of 3 years from OTT, regular menstrual recovery was reported in all patients, and FSH levels ≤16 IU/L were documented in 8/13. Spontaneous pregnancy and live birth rate were 38% and 23%, respectively. After OTT, the live birth rate after IVF was 38.4%, with at least one live birth in 30.7% of OTT patients.

Poirot et al. prospectively evaluated 25 patients, including 16 HL and 7 NHL, who had OTC either more than three months from their last CT cycle or during CT for disease progression or emergency treatment [33]. Ovarian tissue harvesting was performed with laparoscopy and OTT, as carried out after approval from hematologists and histological confirmation of the absence of cancer infiltration in fragments of the cryopreserved cortex. The fragments were then transplanted orthotopically in the ovarian cortex, and ovarian function was monitored with scans and blood sampling for hormone levels. Ovarian function recovery (OFR) was defined by the occurrence of menstruation. Age at OTC and OTT ranged from 19 to 31 and 24 to 40 years, respectively. Patients requesting OTT had premature ovarian failure (POF). After a median follow-up of 32 months since OTT, the cumulative incidence of OFR was 91.3%, and the median time to recovery was 4.6 months. The technique was effective, and 11 out of 23 patients (47.8%) had 20 pregnancies and had at least 1 live birth each. Biases in the previous two studies may have come from the small sample size and the heterogeneous type of included patients.

Cryopreservation of mature oocytes, a technique available for postpubertal females, is another widely offered option to patients starting gonadotoxic treatment. Delay in treatment initiation is the major concern associated with this procedure due to a relatively long time needed for ovarian stimulation. However, in a prospective observational study by Viviani et al. of 19 newly diagnosed HL patients who were offered cryopreservation of mature oocytes before CT, the average time measured from the first fertility preservation consultation and CT start was as short as 22 days [34]. Eligible patients were between 18 and 39 years old, and a random start protocol was used for ovarian stimulation. The definition and representativeness of the population were judged appropriate, and the follow-up was adequate for the outcome; however, the sample size was small.

Data on the proportion of patients requesting the use of cryopreserved oocytes remain scarce. In a retrospective study by Specchia et al., 1 out of 66 patients (1.5%) who cryopreserved mature oocytes requested the oocytes for subsequent IVF, but the follow-up period was not clearly stated [35]. The second cohort study by Hyman et al. evaluated the efficiency of 56 oocyte retrieval cycles for fertility preservation in HL and NHL, where 31 patients underwent stimulation and retrieval of mature oocytes and 25 patients underwent immature oocyte retrieval for subsequent IVM and mature oocyte/embryo cryopreservation [36]. The analysis showed a similar number of mature oocyte and/or embryos to freeze between the two groups. This suggests that immature oocyte collection and IVM, which is still an experimental procedure, may offer a faster alternative to mature oocyte collection, but larger prospective studies are needed to confirm this observation. However, no data on subsequent pregnancy rates were reported. Bias may arise from the unclear selection of the population studied. No data in the literature could be retrieved regarding the cryopreservation of immature oocytes.

### 3.7. PICO 3. Is the Use of Gonadotropin-Releasing Hormone Analogs (GnRHa) during Treatment Effective in Reducing the Risk of Infertility in Adult HL or DLBCL Premenopausal Female Patients Receiving CT Regimens Containing or Not Containing Alkylating Agents or HD-CT or RT to Pelvic Nodes?

**Figure 3:**
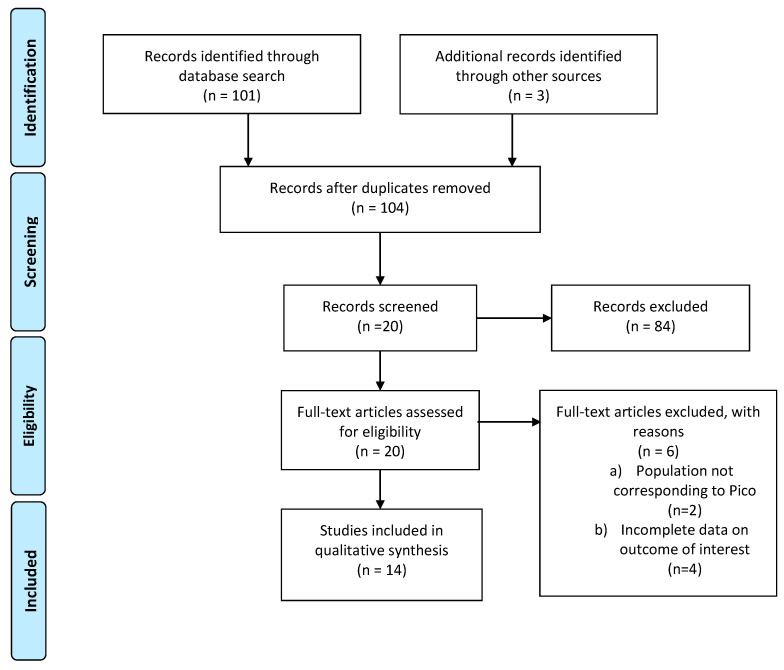
PRISMA flow-chart on GnRH analogs to reduce infertility risk in HL or DLBCL patients.

The literature search yielded 104 studies after duplicates were removed and the revision of titles and abstracts; 19 studies were identified as potentially eligible for inclusion in this analysis. As detailed in Figure 3 (PRISMA flow-chart PICO 3), data with effect estimates (incidence, RR) of menstruation recovery or maintenance, spontaneous pregnancies, and hormonal evaluation could be extracted from 12 publications, all of which were published in peer-reviewed journals between 1990 and 2020, as detailed in Table S3.

#### 3.7.1. Maintenance or Recovery of Menstruation

The effect of GnRHa (goserelin or triptorelin) given in combination with CT versus CT alone on the outcome of menstruation maintenance or recovery or on the opposite outcome of POF was evaluated in three randomized trials (RCTs), one metanalysis, and one case–control study. In addition, we summarized a number of prospective nonrandomized studies using concurrent and historical controls and retrospective studies. It should be noticed that POF has not been uniquely defined. However, the studies widely used the lack of recovery of menstrual cycles and postmenopausal FSH levels (≥40 IU/L) as POF, while others used only FSH serum levels.

Of the three randomized trials, Loverro et al. examined 29 HL patients with a median age of 24 years at the time of treatment and in complete remission (CR) for at least three years after having received CT with or without RT (6 × ABVD: n = 13; 6 × ABVD/COPP: n = 13; 6 × COPP/ABVD—DHAP: n = 3; supradiaphragmatic RT: n = 24) [37]. No patient received pelvic RT. Before starting CT, patients were randomly assigned to receive triptorelin (3.25 mg) monthly or depot (11.25 mg) every three months in concomitance (n = 14) or not (n = 15). Amenorrhea occurred more frequently in the non-GnRHa group than in the GnRHa group (46% versus 0%), suggesting a possible protective effect of GnRHa. Authors cautioned, however, that the observational period after CT was significantly longer in the non-GnRHa group, and, therefore, the appearance of POF could be detected at a later time point in the GnRHa arm. The study also observed that the incidence of amenorrhea varied between patients aged less than 30 years compared with those over 30 years. We judged this study based on the ROB scale for RCT as at unclear risk of bias due to unclear random sequence generation, allocation concealment, and blinding, which were all not mentioned. The protocol was not mentioned. Nevertheless, the study population was well described, and all women were followed-up and included in the analysis. We, therefore, judged the study to be at low risk of bias on incomplete outcome data and selective reporting bias.

The second randomized trial conducted by the GHSG was prematurely closed after an unplanned interim analysis due to slow enrollment and a much lower ovarian protection rate with GnRHa than the 20% rate assumed. The authors conducted the analysis on 23 patients, aged 18 to 40 years, diagnosed with advanced-stage HL and treated with 8 cycles of eBEACOPP [38]. Patients were randomly allocated to receive CT with either oral contraceptives (levonorgestrel 0.15 mg + ethinyl–estradiol 0.03 mg) (Arm A) or monthly goserelin (3.8 mg) (Arm B). The authors defined POF on the basis of menstrual status after a median of 18 months from the end of CT and FSH serum levels. Regular menstrual cycles after CT were more frequently reported in Arm B than in Arm A (7 of 10 vs. 3 of 9). Twelve months after the end of CT, FSH returned to normal values in 22% and 30% of patients in Arms A and B, respectively. We rated this study as at high risk of bias for attrition because the study was stopped early, and only 23 women were included in the final analysis. We judged the trial to be at unclear risk of bias for randomization and blinding because the authors did not mention how the randomization was performed or whether the participant and outcome assessment was blinded or not. The risk of selective reporting was considered low because reporting outcomes were consistent between the sections of the study.

The third randomized trial was conducted by Demeestere et al. on 129 HL or NHL (subtypes not specified) premenopausal women aged 18 to 38 years. The patients received front-line or salvage CT regimens that were either associated with low gonadal toxicity risk (8 cycles of ABVD, R-CHOP, or R-CHOEP (cyclophosphamide, doxorubicin, vincristine, etoposide, prednisone)) or high gonadal toxicity risk (BEACOPPesc, ACVBP (doxorubicin, cyclophosphamide, vindesine, bleomycin, prednisone), followed by consolidation with methotrexate, etoposide, ifosfamide, and cytarabine BEAM conditioning regimens, followed by ASCT) [39,40]. Patients in the control group received only norethisterone acetate (5 mg) daily, starting 10 days before CT start (n = 39), while patients in the GnRH-a group received the same treatment plus triptorelin (11.25 mg) every 12 weeks (n = 45). The authors defined POF on the basis of at least one episode of FSH serum level ≥40 IU/L, and ovarian function recovery was defined as FSH ≤ 10 UI/L. After one year of follow-up from the end of CT, no significant difference between the two groups was reported in terms of POF or ovarian function recovery. The updated analysis of 67 evaluable patients after a median follow-up of 5 years showed that the coadministration of GnRH-a during CT did not influence the risk of POF (19.4% vs. 25%; *p* = 0.763; odds ratio, 0.702, 95% CI, 0.15–3.24; *p* = 0.651) [39]. Based on the ROB/RCT scale, we judged the study at high risk of bias for attrition because 30.7% of patients in the GnRHa group and 39% in the control group dropped out during the study. We rated the study at low risk of bias for randomization because a random allocation sequence was generated and allocation concealment was implemented using central telephone or fax systems; however, the study was at unclear risk for blinding, which was not mentioned. The Demeestere trial was stopped for futility after 129 patients were enrolled, and the interim analysis on 99 patients showed low and superimposable POF rates in the GnRHa and control groups. The study initially planned to enroll 157 patients based on the expected difference in ovarian recovery rates of 20% to 25%. The planned follow-up time was only one year, which is not sufficient to exclude a higher rate of POF afterward, although the final unplanned analysis was updated after 5 years of follow-up.

In a meta-analysis of 12 RCTs on the adjuvant role of GnRHa for the prevention of POF, 1369 premenopausal women with breast cancer, ovarian cancer, or HL (4 studies included), aged between 12 and 51 years, were studied and reported with a recent update [41]. A higher incidence of menstruation recovery or maintenance was observed in the GnRHa cotreatment group (74.5%) compared to the CT-alone group (50%) during a follow-up period of ≤12 months (RR 1.6, 95% CI, 1.14–2.24; *p* = 0.006). However, this difference did not persist with a longer follow-up period (72.9% vs. 65.4%; RR 1.08, 95% CI, 0.95–1.22). GnRHa appeared to be effective in reducing the incidence of treatment-related POF (10.7% vs. 25.3%; RR 0.44, 95% CI, 0.31–0.61; *p* < 0.00001) and in preserving ovulation, whose incidence was 61.7% vs. 25% in the GnRH-a group and in controls, respectively (RR 2.47, 95% CI, 1.43–4.26; *p* = 0.001). Based on an AMSTAR2 scale evaluation, the meta-analysis was judged as a high-quality systematic review.

Moving to observational studies, the outcome of amenorrhea was evaluated in a case–control study conducted on a small patient population of 10 HL patients with GnRHa (monthly leuprolin 3.57 mg or goserelin 3.6 mg) treated between 2001 and 2007 with 2–4 cycles of ABVD or OPPA (vincristine, procarbazine, prednisone, doxorubicin), followed by COPP or BEACOPP, matched with 31 patients treated with the same chemotherapy regimens without GnRHa cotreatment and with 10 additional healthy women treated for severe male factor infertility [42]. Patients were aged between 17 and 35 years and were treated between 2001 and 2007; only two patients, one in each group, who received standard-dose BEACOPP every 14 days experienced amenorrhea, whereas all other patients were still regularly menstruating. Therefore, the study failed to document any significant difference in POF after at least 6 months of follow-up from the end of CT. Based on NOS, the quality of this study was, in general, considered intermediate: the patients were exactly matched for age and treatment, the selection and exposure of controls were good, and a bias could be represented by the record linkage of the case definition. However, the major limitation of this study was the small number of evaluable patients treated with different CT regimens at different risks of gonadal toxicity.

Another prospective observational study was conducted on 78 patients with hematologic malignancies, including HL, NHL, and other non-neoplastic diagnoses treated between 1998 and 2007 with different CT regimens. Concomitant monthly triptorelin 3.75 mg ± estrogen-progestin given to 33 patients was compared to only estrogen-progestin in 45 controls [43]. No significant difference in amenorrhea rates in the two groups was seen (OR 0.52; CI 95%, 0.21–1.33, *p* = 0.172). We judged this study as low quality due to the bias represented by inclusion in the treatment or control group based on the women’s personal choice and the small patient population with different hematological malignancies and non-neoplastic diseases.

Similarly, in another retrospective study of HL female patients receiving frontline CT (n = 54), mainly ABVD or salvage CT, including high cumulative doses of alkylating agents (n = 7), no significant reduction in POF could be documented despite the use of GnRH-a. [44]. All seven relapsed or refractory patients became amenorrheic despite the use of GnRH-a. The study did not include any control group. The quality of the study was, in general, intermediate: the selection of patients was good, the comparability was intermediate, and the assessment of outcome, which was based on record linkage, was also intermediate; one further bias was represented by the 21% of patients lost to follow-up.

On the contrary, a significant difference in the rate of POF with the use of GnRHa was reported in several prospective nonrandomized studies with concurrent and historical controls. For example, in 115 HL patients after frontline therapy, the POF rate was 3.1% and 37% in the GnRH group (n = 65) and the control group (n = 46), respectively (*p* < 0.001) [45]. This difference remained significant in the subgroup of patients treated with alkylating regimens such as BEACOPP or monthly alternating MOPP/ABVD. In these patients, the administration of GnRHa (n = 40) has been effective in avoiding POF (5%) compared to 44% in patients not receiving the GnRHa cotreatment (n = 36) (*p* < 0.001). In contrast, no significant difference in the POF rate (0% vs. 10%, *p* = NS) was detected among patients receiving ABVD. We judged this study by NOS to be, in general, of good quality, in particular with regard to selection and adequacy of follow-up. Furthermore, for comparability, an analysis of variance was performed to evaluate whether there was a difference between study groups and the various demographic parameters.

Castelo-Branco et al. observed a POF rate of 10% vs. 23% (*p* < 0.05) after monthly 3.75 mg triptorelin + daily tibolone in 30 HL patients vs. none in the 26 controls. These 56 HL patients were treated with MOPP/ABV(D) ± RT (n = 5 vs. 7), or ABVD ± RT (n = 20 vs. 17) or salvage therapy + ASCT (n = 5 vs. 2) [46]. The study was, in general, of low quality: bias could be detected in comparability, in the assessment of outcome based on record linkage, and in the absence of a definition of the length of follow-up.

Another positive study demonstrated a reduction in the rate of POF after a median follow-up of 7 years, from 82% to 33% (*p* = 0.023), in female lymphoma patients who received monthly 3.75 mg triptorelin in conjunction with conditioning CT and ASCT (n = 21) compared with CT and ASCT only (n = 11) but not in leukemia patients (GnRHa group, n = 20 vs. CT + SCT only, n = 12: 90% vs. 92%) [19]. The quality of this study (NOS) was globally good: the representativeness, selection, and exposure were good as well as the duration of the follow-up; the assessment of the outcome was based on record linkage, and the information about pretransplantation CT could be retrieved for 65% of the cohorts.

The protective effect of GnRHa therapy on POF was also confirmed in another retrospective cohort study conducted by Blumenfeld et al. that documented the POF rate of 13% vs. 51% (*p* = 0.0001) in 286 patients who received the GnRH-agonist cotreatment compared to 188 patients treated with CT alone [47]. This study included 89 HL patients treated with ABVD, BEACOPP, or eBEACOPP and also ASCT; 32 not-otherwise-specified NHL patients who had received CHOP or CVAD (cyclophosphamide, vincristine, dexamethasone, methotrexate, cytarabine) or ASCT; 34 acute leukemias and 42 patients with other nonhematologic diseases, including breast cancer, ovarian cancer, sarcoma, aplastic anemia, myelodysplasia, and immune diseases. We could not rule out that the patient population of this study was at least partly presented in the earlier work by the same authors. The quality of this study (NOS) was globally good: the representativeness, selection, and exposure were good as well as the duration of the follow-up.

#### 3.7.2. Hormonal Evaluation of Ovarian Function (FSH, Estradiol, AMH Serum Levels)

Seven studies were included to evaluate hormone levels in response to our PICO: three RCTs, one meta-analysis, and three observational studies. As mentioned before, Loverro et al. found no significant difference in serum hormonal levels of FSH, inhibin B, AMH, and the number of antral follicle count (AFC) between the two groups of HL patients receiving or not the GnRHa cotreatment [37]. However, the mean time ± SD from the end of therapy to hormonal evaluation was significantly longer in the control group compared with the GnRHa group (5.93 ± 4.47 vs. 2.42 ± 1.7 years; *p* = 0.0541).

In the RCT conducted by the GHSG, hormonal evaluation included the assessment of FSH, estradiol, and AMH at baseline, during CT, and 6 and 12 months after the end of treatment [38]. The GnRHa cotreatment had no protective effect on ovarian reserves after an aggressive highly gonadotoxic CT regimen such as eBEACOPP. 

Similarly, in the third RCT conducted by Demeestere et al. [39,40], AMH levels were available in only 31 patients after one year of follow-up. Initially, values greater than 1 ng/mL were more frequently detected in the GnRHa group than in the control group (8/16 vs. 2/15; *p* = 0.023), and the mean AMH values were higher in the GnRHa group than in the control group (1.40 ± 0.35 vs. 0.56 ± 0.15 ng/mL; *p* = 0.040). By extending the follow-up, no significant difference was reported in the risk of low FSH values among the two groups (*p* = 0.434), and similar rates of low AMH levels (<0.5 ng/mL) were observed in both groups after two to four years of follow-up. It should be noted, however, that only a small patient population had AMH available at least once during follow-up, and, therefore, this study cannot provide strong evidence of the effect of GnRHa cotreatment based on serum hormonal levels.

The assessment of the quality of these randomized controlled trials has been previously reported.

Consistent with these results was also the meta-analysis conducted by Chen et al., previously described [41], which pooled the results of two studies and found that there was no difference in FSH levels between the CT plus GnRHa group and the CT-alone group in 71 patients (SMD 0.26, 95% CI, −0.80–1.31; *p* = 0.63). No difference was also documented in the AMH levels between the two groups (SMD −0.05, 95% CI, −0.78–0.68; *p* = 0.89).

Only two studies found that GnRHa had a protective effect on ovaries with respect to estradiol levels, whereas five other studies found no difference in estradiol levels between groups. It should be stressed that only 1 out of 10 listed studies included lymphoma patients. As already mentioned, the quality of this meta-analysis by the AMSTAR tool was high, also with regard to the outcome of hormonal evaluation.

Three observational studies, already mentioned in the previous section, reported sufficient data on hormonal levels after CT. Nitzschke et al. showed no significant difference in hormonal parameters in HL patients with and without GnRHa cotreatment: the mean FSH serum levels were 14.7 (SD 6.6) and 14.5 U/L (SD 0.9), respectively, significantly higher compared to healthy women of a control group (5.3 U/L ± SD 1.8); the mean AMH levels were 1.6 (SD 1.4) and 1.4 ng/mL (SD 1.1), respectively, significantly lower than in healthy women (3.9 ng/mL ± SD 1.2) [42]. 

Castelo-Branco et al. described a significantly lower mean FSH level and a significantly higher estradiol level in the GnRHa group than in the control group at the final evaluation (FSH: 14.91 ± 18.65 vs. 79.85 ± 45.83, *p* = 0.001; estradiol: 59.74 ± 53.78 vs. 25.71 ± 13.01, *p* = 0.05), but no clear follow-up period was mentioned [46].

In the third observational prospective study by Huser et al., 108 HL patients, treated between 2005 and 2010 with different CT regimens (6–8 cycles of eBEACOPP or 2 cycles of ABVD or 2 cycles of eBEACOPP, followed by 2 cycles of ABVD) also received monthly triptorelin injections and had a mean FSH level of 11.6 ± 17.9 IU/L after one year from the end of treatment [48]. After two years of follow-up, a diminished ovarian reserve, defined by FSH level >15 IU/L, occurred in 10 patients only (9.3%), with average FSH levels of 46.0 ± 21.5 IU/L. The quality of this study by the NOS tool was globally intermediate as the derivation of the nonexposed cohort was not mentioned, the assessment of outcome was based on record linkage, and the duration of the follow-up was short, only two years, whereas the representativeness and exposure were good.

#### 3.7.3. Pregnancy

Post-treatment pregnancies are explored in a few studies. Some of these studies have already been previously described in the previous two outcomes.

Based on the three RCTs, no significant difference could be detected in any study between patients treated with GnRHa combined with CT compared to CT only. Loverro et al. reported only two pregnancies after cancer therapy, both in the group without the GnRHa cotreatment; however, the observation period after CT was longer in this group [37].

No pregnancy occurred in HL patients receiving the eBEACOPP regimen with or without GnRHa administration [38].

Demeestere et al. reported a high pregnancy rate in both the GnRHa and control groups (15/35 (53.1%) and 17/32 (42.8%); *p* = 0.467) after a median follow-up of 5.33 years [40].

In the meta-analysis by Chen et al., the incidence of pregnancy was 32 out of 356 (9%) in the GnRHa plus CT group and 22 out of 347 (6.3%) in the CT-alone group [41]. The RR was 1.59 (95% CI, 0.93–2.7), with no difference between groups.

No significant difference in pregnancy incidence was reported in two prospective observational studies. In the Blumenfeld Z. et al. study, HL or NHL patients receiving concomitant GnRHa or not had a pregnancy rate of 40% (26/65) vs. 43.5% (20/46) [45]. Similarly, the Driul L. et al. study on HL, NHL, AL, and AML patients showed a rate of 6% (2/33) vs. 4% (2/45), respectively, with an OR for spontaneous conception of 1.39 (95% CI, 0.19–10.39; *p* = 0.750: 6% (2/33) vs. 4% (2/45) [43].

On the contrary, a benefit of the administration of GnRHa during CT was reported by Blumenfeld et al. in a retrospective comparison of an observational cohort with a historical control group [47]. A total of 65% of 81 patients receiving GnRHa conceived compared to 37.9% of 66 patients treated with CT only (*p* = 0.0004); the OR for spontaneously conceiving was 3.12 for the patients who received GnRHa combined with CT versus those who received CT without GnRH-a (95% CI, 1.7–5.8). Furthermore, in the GnRHa group, 16% of patients spontaneously conceived despite receiving CT after age 30 compared to only 7% in the same age group in controls. This difference, however, could not reach a significant level due to the small number of patients in each group.

### 3.8. PICO 4—What Are the Most Appropriate Fertility Investigations in Male and Female HL or DLBCL Patients Treated with CT and/or Pelvic (Inguino-Iliac) Irradiation?

**Figure 4:**
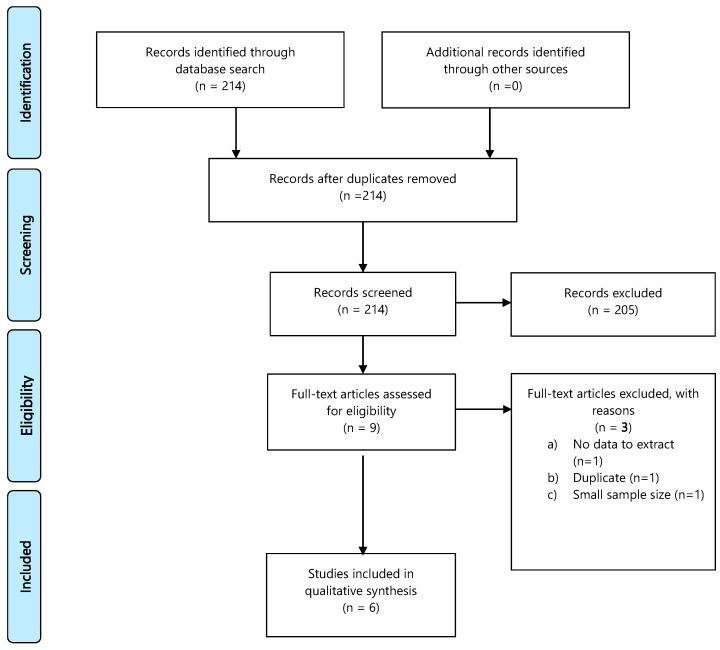
PRISMA flow-chart on appropriate fertility investigations in HL or DLBCL patients.

A database search identified 214 records; 205 publications were excluded, 9 full-text articles were assessed for eligibility, and 6 were included in the final analysis. Details of the whole screening process, including reasons for full-text exclusion, are reported in Figure 4 (PRISMA flow-chart PICO 4) and Table S3.

### 3.9. PICO 4a. What Are the Most Appropriate Fertility Investigations in Male HL or DLBCL Patients Treated with CT and/or Pelvic (Inguino-Iliac) Irradiation?

We extracted data from Behringer et al.’s cohort of 761 male survivors who were enrolled in the GHSG HD13-HD14-HD15 trials [15]. The patients were in CR for at least one year after CT, the age at assignment was 19–49 years, and the upper age limit at fertility assessment was 57 years. Hormone levels differed with stage of disease, number of cycles, and intensity of CT regimen. In particular, the inhibin-B/FSH ratio was >23.5 ng/U; an indicator of fertility [49] was only seen after ABVD or 2 ABVD + 2 eBEACOPP (2 + 2). The reported spontaneous conception rate was 10.5% after ABVD, 3.5% after 2 + 2, and 1.5% after 6 cycles of eBEACOPP; no conception occurred after 8 cycles of eBEACOPP or 8 cycles of standard BEACOPP. Testosterone levels remained within a normal range after all treatment regimens, indicating its non-usefulness in investigating gonadal toxicity after CT. The length of follow-up and the size and representativeness of the sample studied were judged as adequate for the outcome considered.

Kulkarni et al. prospectively analyzed a small sample of 38 HL men aged 15–43 years who were treated with ABVD (n = 26), MOPP/ABVD (n = 11), and MOPP/ABVD/COPP (n = 1) [50]. After a mean observation time from the end of treatment of 34 months (range, 12–68), the median sperm count was 63.4 × 10^6^ in the ABVD group. Azoospermia was reported in only 4% of ABVD-treated patients compared to 91% of patients also receiving the alkylating MOPP or COPP regimen (*p* < 0.001). FSH levels were significantly higher in the COPP/ABVD group, whereas no significant difference in testosterone levels was reported. Three patients who belonged to the ABVD group fathered children. The length and completeness of the follow-up were good, but biases could have arisen from the unclear selection and the small sample size of the study. 

### 3.10. PICO 4b. What Are the Most Appropriate Fertility Investigations in Female HL or DLBCL Patients Treated with CT and/or Pelvic (Inguino-Iliac) Irradiation?

Anderson et al. prospectively studied the changes in AMH and FSH blood levels in 67 female patients treated according to the RATHL trial, as previously described in the PICO 1 results section [18]. The study was not designed to investigate fertility but proved AMH as a good biomarker of toxic effects of different CT regimens for HL on ovarian reserves. The evaluation of the quality of the study has already been reported.

Behringer et al. conducted an observational study on 263 female patients with early-stage HL, aged 18 to 40 years, who were enrolled in the HD 14 trial. The trial arms consisted of 4 ABVD cycles (Arm A) and 2 eBEACOPP cycles followed by 2 ABVD cycles in Arm B [16]. A significant difference in AMH levels was observed between the two arms in favor of ABD in both younger patients aged 18 to 29 years, (Arm A: 2.2 ng/mL versus Arm B: 0.9 ng/mL) as well as older ones aged 30 to 45 years (Arm A: 0.8 ng/mL versus Arm B: 0.03 ng/mL). FSH levels were lower in Arm A than in Arm B but only in older women (FSH age Arm A: 4.4 IU/L versus Arm B: 11.9 IU/L). No significant difference in regular menstrual cycles was observed between the treatment arms nor time to regular menstrual cycle after CT. However, a slightly lower incidence of normomenorrhea was reported among older patients treated with eBEACOPP (Arm A vs. Arm B: 84% vs. 74%).

As previously reported, among the 562 patients enrolled in HD13–15 GHSG trials, significant differences in AMH and FSH serum levels were documented in favor of less intensive therapy for both age groups [15]. The study also considered older age at the time of treatment and an interval to menstrual recovery longer than 12 months as risk factors for ovarian POF. The quality of this study has already been mentioned. A similar pattern was seen in a study by Decanter et al., in which AMH was correlated with CT-induced gonadotoxicity, but the age effect was excluded because the age of the 30 analyzed patients ranged from 18 to 32 years [51]. AMH levels were measured before CT start (AMH0), 15 days after the first CT cycle (AMH1), 15 days before the last CT cycle (AMH2), and every 3 months after the end of CT (AMH+3n). In patients treated with ABVD, AMH dropped from AMH0 to a minimum at AMH2 (AMH0 vs. AMH1: *p* < 0.05; AMH0 vs. AMH2: *p* < 0.05), then rose continuously to AMH0 levels at AMH12. In patients treated with non-ABVD CT, AMH dropped rapidly from AMH0 to a minimum at AMH2 (AMH0 vs. AMH 1: *p* < 0.0001), and, despite a slight rise, the AMH12 levels remained lower than AMH0. After the end of chemotherapy, the rise in AMH concentrations was much pronounced in the ABVD group, with significant (*p* < 0.05) differences between the two groups for AMH+3, +6, +9, and +12. The study had a prospective design and was judged by the NOS tool as adequate for selection and completeness of follow-up. Bias may be derived from the sample size.

Loverro et al. investigated a cohort of 29 female patients with HL treated with CT with or without RT (6 ABVD: n = 13, 6 ABVD/COPP: n = 13, 6 COPP/ABV: n = 3; supradiaphragmatic RT: n = 24) [37]. All patients were in CR for at least three years at the time of inclusion; the mean age at diagnosis was 24.3 years, and the mean age at assessment was 28.5 years; the mean time between end of CT and study evaluation was 4.2 ± 2.8 years. The investigation was based on gynecological examinations, records of menstrual pattern, obstetric histories, AFC, serum FSH, LH, inhibin-B, and AMH determinations; 21 out of 29 women (72%) showed regular menstrual cycles, but only two of them (10%) became pregnant after treatment. The best predictive value for reproductive outcome came from the combination of AMH and AFC (sensitivity 83%, specificity 88%). The study has been judged by NOS as adequate for selection, follow-up length, and completeness of follow-up. Bias may come from the size of the sample.

### 3.11. PICO 5—What Is the Best Interval to Respect from End of Treatment Before Conceiving?

**Figure 5:**
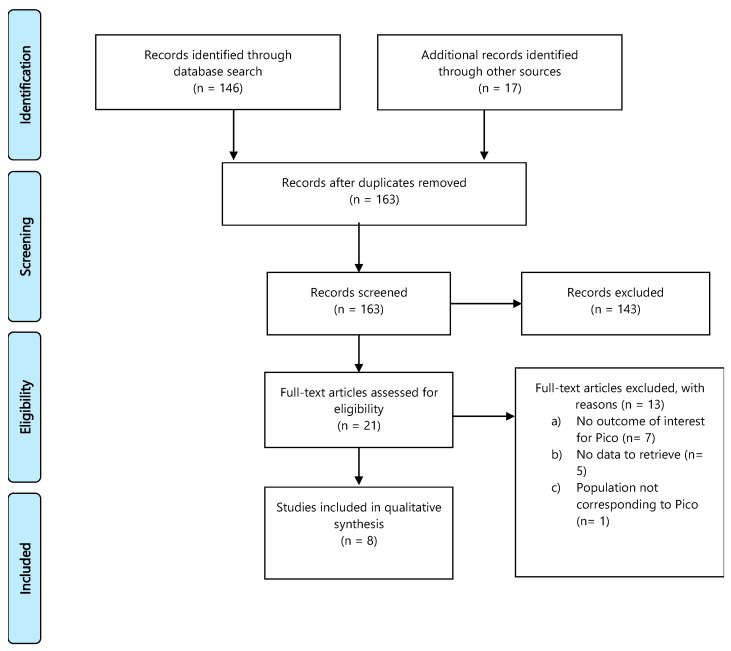
PRISMA flow-chart on best interval from end of the treatment for HL or DLBCL to conception.

The literature search yielded 163 studies with duplicate removal. After reviewing titles and abstracts, 21 studies were identified as potentially eligible for inclusion. After a full-text review, 8 studies were included in the final sample for relative analysis (Table S3). Details of the whole screening process, including reasons for full-text exclusion, are reported in Figure 5 (PRISMA flow-chart PICO 5).

In regard to this specific PICO, no RCTs reported were found and only eight retrospective observational studies on HL and DLBCL survivors reported the number of spontaneous pregnancies, live births, miscarriages, stillbirths, and congenital abnormalities as PICO outcomes. However, none of the studies examined the optimal timing of conception after the end of antitumoral treatment.

Aisner et al. analyzed data from 43 HL female patients who were actively attempting conception and treated by a single institution between 1965 and 1985, utilizing questionnaires on pregnancy frequency and outcome [52]. HL treatment was either RT alone (37%), CT alone given with MOPP or COPP regimen (9%), or a combined modality (CT + RT: 54%); among patients receiving RT ± CT, pelvic node RT was delivered to 23% of them. In total, 35 females achieved 54 pregnancies, resulting in 42 children, 1 spontaneous abortion, 9 elective abortions, and 2 stillborn births. The pregnancy rate did not differ by therapy, and the median interval from the end of therapy to the date of first childbirth was 5.5 years (range, 8 months to 14 years). Therefore, their recommendation was to delay attempts of pregnancy for at least one year after treatment. There was no apparent increase in pregnancy complications, spontaneous abortions, or major birth defects compared with pregnancies prior to treatment or to the general population. The quality of the study was, in general, intermediate: ascertainment of exposure was based on structured interviews, and measurements of outcomes and the duration of follow-up were adequate; however, no description of the nonexposed cohort was provided.

Pregnancy rates at 12 months from the first attempt to conceive were assessed in an observational interview-based single-center study conducted by Hodgson et al. [53]. The study included 36 HL female patients who were treated between 1983 and 1999 with ABVD (2–4 cycles: 50%; 4–6 cycles: 44%; >6 cycles: 6%) without pelvic node RT. Patients were enrolled if they were alive without relapse after at least 3 years from the end of therapy and were compared with 29 healthy friend or sibling controls. The pregnancy rates were similar between HL patients and control groups (70% and 75%, respectively), with a fertility ratio of 0.94 (95% CI, 0.53–1.66, *p* = 0.84). These results indicate that HL patients in CR for at least three years from the end of front-line treatment do not experience significant infertility. The quality of this study was good: selection of cases, measurement of outcome, and duration of follow-up were good. 

Kiserud et al. reported the outcome of post-treatment parenthood using a questionnaire sent to HL survivors who had been treated between 1971 and 1998. Patients were aged below 40 years at diagnosis and were treated with RT alone or low-, medium- and high gonadotoxic CT regimens alone or in combination with RT [8]. No difference was observed in the 10-year probability of parenthood by gender, with 75% of females and 63% of males having offspring. However, rates were significantly different by age groups, namely, ≤20, 20–30, and ≥30 years in females, and by various treatment groups in both genders in terms of RT only vs. low-risk CT, medium-risk CT, and high-risk CT (71% vs. 85% vs. 35% vs. 18% in males, and 82% vs. 55% vs. 51% vs. 27% in females). The quality of this study was good: selection of cases, measurement of outcome, and duration of follow-up were good; the only bias was represented by the 11% of patients lost to follow-up.

The EORTC and GELA groups used a questionnaire to investigate mother- and fatherhood in a large cohort of 1654 long-term HL survivors from 9 consecutive trials conducted between 1964 and 2004 and compared them with 6414 matched controls from the general population [54]. The likelihood of conception after treatment was significantly reduced in HL survivors compared to the general population. The OR for having children after treatment was 0.77 (95% CI, 0.68–0.87; *p* < 0.001) for cases compared to controls, with different outcomes among females and males, the OR being 0.64 (95% CI, 0.52–0.77; *p* < 0.001) for females and 0.84 for males (95% CI, 0.70–1; *p* = 0.06). Among the 666 survivors who attempted post-treatment parenthood, the success rate was 77%, without a significant difference between males and females, despite a more frequent use of ARTs in males. Among males attempting post-therapy parenthood, 63% (217 out of 345) succeeded spontaneously, and a further 15% (n = 49) succeeded with the help of ARTs, whereas the corresponding figures in females were 74% (295 out of 400) and 2% (n = 8), respectively. Factors associated with a lower probability of spontaneous post-therapy conceptions were age ≥35 years (OR, 0.16; *p* < 0.001), ≥3 MOPP-equivalent cycles (OR, 0.04; *p* < 0.001), and salvage therapy (OR, 0.10; *p* < 0.001) for males and age ≥35 years (OR, 0.13; *p* < 0.001), ≥3 MOPP-equivalent cycles (OR, 0.51; *p* = 0.01), and salvage therapy (OR, 0.27; *p* < 0.001) for females. The study did not address the interval between HL treatment end and conception. Based on NOS, we judged the quality of this study as good: selection of cases, measurement of outcome, and duration of follow-up were good.

In a Swedish-Cancer-Register-based study, time to first childbirth was estimated by comparing HL relapse-free female survivors treated between 1992 and 2009 with healthy matched controls from the national population-based registry [55]. For limited-stage HL, standard treatment consisted of two to four MOPP-ABV cycles until 1996, then two to four cycles of ABVD afterward; patients with advanced-stage were first offered six to eight cycles of MOPP-ABV and then only six to eight cycles of ABVD. The follow-up was restricted to the first seven years after diagnosis to avoid different lengths of follow-up between calendar periods. HL patients had a lower childbirth rate compared to controls (HR, 0.78; 95% CI, 0.63–0.97), mainly in the first three years of follow-up (HR, 0.58; 95% CI, 0.41–0.81), whereas after three years, there was no significant difference in childbirth rates between patients and controls. Among the 449 HL patients, 101 (22.5%) had a childbirth (rate, 57.2 per 1000 person years), which was 28.9% (rate, 70.1 per 1000 person years) among 2210 compared controls. A lower rate was observed in patients diagnosed between 1992 and 1997, whereas patients treated from 2004 to 2009 had similar childbearing potential to the Swedish general female population (HR, 0.93; 95% CI, 0.66–1.33). Even patients treated with the most gonadotoxic regimen, BEACOPP, showed reduced conceptions during the first three years of follow-up but approached the levels of compared controls later on. The quality of this study was, in general, good: selection of cases, measurement of outcome, and duration of follow-up were adequate.

Gaudio et al., in a retrospective study, examined the pregnancy rate and time to pregnancy among 89 HL women, ≤50 years of age, who were in CR and alive without relapse after at least one year from the end of treatment [56]. Patients were treated between 2006 and 2015; 83% received front-line therapy with the ABVD regimen, and 17% also received salvage treatment with IGEV (ifosfamide, gemcitabine, vinorelbine, prednisone) (13/15) and BEACOPPesc (2/15); 13 out of 89 patients (15%) underwent ASCT. Eight patients (9%) had a pregnancy; all but one were treated with front-line ABVD after a median time from the end of therapy to pregnancy of 50 months (range, 35–72). The cumulative incidence of pregnancy at 70 months follow-up was 11%. No further data on pregnancy outcomes or malformation defects were reported. The quality of this study was good: selection of cases, measurement of outcome, and duration of follow-up were good.

Two studies, on the other hand, focused only on patients who underwent hematopoietic stem cell transplantation. In the first study, Carter et al. investigated self-reported pregnancy occurrence and pregnancy outcomes in transplant survivors who were treated between 1974 and 1998, were alive at least two years from transplant irrespective of disease status, and participated in the Bone Marrow Transplant Survivor Study (BMTSS). Patients’ median age at transplant was 33.3 years (range, 21–45); 29.9% of them had received a transplant for HL or NHL and were compared to a sibling group [57]. After a median follow-up of 7.7 years (range, 2–24.4), 34 (5.5%) survivors (26 males and 8 females) reported 54 conceptions. In ASCT recipients, 8 males and 4 females each had nine conceptions after a median of 4 years from transplant. Among the allo-SCT recipients, 18 males and 4 females reported 31 and 5 post-transplant conceptions, respectively, after a median of 3.6 years from the transplant. Transplant survivors had a 36-fold increased risk of not reporting a conception compared to the sibling group (OR 35.9, 95% CI, 23.2–55.8; *p* < 0.0001). However, transplant survivors were similar to siblings in reporting a livebirth (OR 2.2, 95% CI, 0.8–6.2; *p* = 0.1), and they were not at an increased risk for miscarriage or stillbirth (RR 0.7, 95% CI, 0.2–2.1; *p* = 0.5). The study did not provide data on the optimal time interval from transplant to conception. The quality of this study was good: selection of cases, measurement of outcome, and duration of follow-up were good.

The second retrospective cohort study was conducted by Akhtar et al. based on questionnaire and interview methods for 89 females treated in Middle East/Arab countries between 1997 and 2012 with ASCT for HL (80%) or DLBCL NHL (20%) [58]. Patients were aged 14 to 40 years at ASCT and were disease-free for at least 24 months after the transplant; front-line therapy consisted of the ABVD or CHOP regimen, while the ESHAP (etoposide, cisplatin, cytarabine, methylprednisolone) regimen followed by BEAM HD-CT was given as salvage treatment; no patient received pelvic RT. In total, 45% of patients tried to become pregnant, and 26 patients (65%) had 50 pregnancies, resulting in 86% live births, 12% miscarriages, and 2% stillbirths. Birth defects were observed in the two live births. The interval between ASCT and the first pregnancy ranged between 5 to 114 months. Despite the lack of a control group and the questionnaire-based methodology, we believe that the quality of the study, given the large number of cases, is good in relation to selection, measurement of outcome, and duration of follow-up.

## 4. Discussion

Patients facing the diagnosis of HL or DLBCL should be informed about the risk of infertility, mainly due to azoospermia in males and reduced ovarian reserve and premature menopause in females. This literature review has evaluated the incidence of infertility among lymphoma survivors, mainly in patients diagnosed with HL and, to a lesser extent, in patients with NHL, which was either DLBCL or unspecified in various studies. The authors of these studies underlined that the risk of gonadotoxicity depends on the type and stage of lymphoma and, consequently, the type and dosage of anticancer treatment as well as age in the case of female patients [7]. Therefore, fertility preservation options should be offered to patients wishing for future offspring, especially those who will receive gonadotoxic treatment that has a subsequent infertility risk greater than 50%. Other fertility preservation candidates are patients undergoing aggressive chemotherapy regimens that are associated with high cumulative doses of alkylating agents and/or hematopoietic stem cell transplantation [10,59]. However, it is reasonable to offer fertility preservation strategies in less gonadotoxic treatments because it is difficult to predict which patients will relapse and, thus, have to undergo treatment with a high risk of infertility, such as ASCT [55]. Although it is possible to use fertility preservation procedures at the time of relapse, in female patients, the time needed to complete such procedures before starting salvage treatment is often lacking, while in males, semen quality may be poorer after first-line treatment and also due to disease-induced causes, especially in the presence of systemic symptoms.

Many attempts to provide increased efficacy to fertility preservation techniques that help cancer survivors have biological children, an important aspect of quality of life, have been made. At the time of the present review, sperm cryopreservation before gonadotoxic therapies is the most efficient and widely available method used to preserve male reproductive potential. There is no upper limit age for male patients when offering semen cryopreservation [60]. Approximately 80% of survivors face a severe impairment of spermatogenesis, and 40% of patients with a wish for children are not able to conceive spontaneously. In this situation, frozen sperm increases their chances of conception through ART, resulting in a pregnancy rate between 33% and 61% [25,26]. The rate of utilization of frozen sperm has been reported to be between 21% [26] and 27% [21].

Ovarian tissue cryopreservation (OCT) is the most promising fertility preservation technique, and it is increasingly offered to patients before and even after gonadotoxic treatment. Potential candidates for OCT are prepubertal girls and patients younger than 35 years who are in urgent need to initiate treatment, with >50% risk of chemotherapy-induced ovarian failure, have no surgical contraindication, and a solid chance of survival [61]. Data are growing about its safety and efficacy; however, little is known about the proportion of patients who actually go for cryopreservation and their actual utilization rate. Despite that the related studies were of a small sample size, prospective records made it possible to better describe the potentials of this technique in female lymphoma survivors. Both OTC and OTT require laparoscopy and/or mini-laparotomy, skilled operators, and a multidisciplinary team; these are the reasons why they are not feasible in every setting. Oncologic safety is to be ruled out before transplantation. Ovarian tissue transplantation is usually performed in patients with no menstrual function recovery and when hormone levels are compatible with the menopausal state. Regardless of the timing of performing OTC in regard to treatment initiation, OTT proved to be effective in increasing the chances of conception, spontaneously and through ART. It also allows menstrual function recovery in more than 90% of cases, with a median time to recovery of about 4.6 months [33]. Spontaneous conception is reported in a minimum of 38% of patients, with a live birth rate of 23%. The live birth rate after ART in OTT patients is 38.4%, ending up with at least one live birth in 30.7% of OTT patients. Larger numbers will confirm whether the OTC/OTT procedure is the best option and if it should be offered widely to the population subjects of the review, providing chances to restore future fertility and endocrine activity. Cryopreservation of mature oocytes is another option largely offered to postpubertal patient candidates for gonadotoxic treatment. Based on studies reporting cumulative live birth rates, the suggested age limit for oocyte cryopreservation is <36 years [61]. The major concern about it has been the delay in treatment commencement due to the length of time of ovarian stimulation, but where facilities and oncofertility teams were available, the average time measured from first consultation to start of chemotherapy was as short as 22 days [34]. Conclusive figures about the utilization rate of cryopreserved mature oocytes were not possible to retrieve. IVM, which may offer a shorter alternative to mature oocyte collection, is also available to patients assigned to gonadotoxic therapies. In small retrospective cohorts, similar numbers of mature oocytes and/or embryos compared to conventional IVF were reported. However, information about the subsequent pregnancy rates could not be verified [36]. Finally, it should be noted that embryo cryopreservation is an established option for fertility preservation endorsed by ESHRE [61]. However, it is not allowed by law for this purpose in many countries, including Italy; moreover, it implies the risk of losing reproductive autonomy in addition to issues about the property of stored embryos. Cryopreserved thawed embryos are used in approximately 23–26% of ART cycles [62,63]. It yields good results, with a reported live birth rate per transfer of 20% and a cumulative rate per patient of 44% in cancer patients [62,63].

The protective role of GnRH analogs on fertility preservation in female lymphoma patients remains controversial [41]. RCT studies and metanalyses have shown that GnRHa addition is not effective in menstruation maintenance or menstruation recovery when patients are exposed to high cumulative doses of alkylating agents and/or procarbazine or ASCT. Furthermore, none of the studies supported better ovarian function measured by FSH and/or estradiol following GnRHa use or better ovarian reserve measured by AMH. It should be acknowledged that the small number of lymphoma patients examined could not power the studies to measure differences in pregnancy rates after CT. In addition, the desire to conceive was not a criterion for inclusion in the studies and follow-up periods were short [37,38,39,40].

However, the use of GnRHa during front-line chemotherapy can improve the rate of menstrual cycle recovery and reduce the risk of vaginal bleeding during thrombocytopenia. It is important to note that GnRHa should not be considered an alternative option for fertility preservation because its use has not been associated with improved pregnancy or live birth rates. Furthermore, long-term studies evaluating GnRH-a safety are still lacking. GnRH-a should be offered if cryopreservation strategies cannot be performed due to time or safety issues or in addition to these in an attempt to reduce the risk of chemotherapy-induced ovarian insufficiency as well as in women who are not interested in having children but desire to preserve their hormonal ovarian function [64]. Taking into account the available evidence of maintenance or recovery of menstruation with GnRH analogs and their acceptable safety profile, it is difficult to expect that new randomized trials with proper design and sample size will be performed. Since GnRH analogs are currently widely used during CT in lymphoma patients, long-term follow-up data are warranted to provide solid evidence not only of their associated reduction of POF risk but also on improvements in conception rates.

The reviewed studies confirmed that sperm count, FSH level, and the inhibin-B/FSH ratio are appropriate tools to investigate fertility in male patients with HL or DLBCL treated with gonadotoxic therapies, showing changes related to stage of disease, number of cycles, and intensity of chemotherapy. Testosterone levels do not seem to be useful in this perspective since they remain within the normal range after different CT regimens.

Serum AMH level was also found to be a reliable biomarker of CT-induced ovarian toxicity during and after treatment in HL. AMH serum levels were decreased during CT, more profoundly after BEACOPP than after ABVD, with a reciprocal increase of FSH. After the end of CT, in ABVD-treated patients, AMH recovery to baselines values was observed within one year, with levels returning to baseline values. In BEACOPP-treated patients, AMH levels remained significantly lower than at baseline. A significant correlation was seen between AMH at baseline and AMH at two years but not with AMH recovery. Additionally, a significant negative correlation was seen between age and AMH recovery at two years from the end of therapy. Age was also related to time to resumption of menstrual activity, and a time longer than 12 months has to be considered a risk factor for POF. After gonadotoxic treatment, the combination of AMH and AFC shows the best predictive value for reproductive outcome, with sensitivity of 83% and specificity of 88%, according to retrospective data by Loverro et al. [37].

Unfortunately, reviewing the literature did not allow us to clarify the optimal time interval from the end of cancer treatment to conception. The current indications in females are to avoid pregnancy in the period of greatest risk of lymphoma recurrence, corresponding to the first two to three years, calculated from the end of treatment. For males, a semen analysis is recommended to exclude serious alterations in spermatogenesis at least one year after the end of treatment before attempting spontaneous conception. The majority of studies have included HL patients who were free of relapse after at least three years from the end of treatment. The interval between completion of CT to pregnancy ranged from 3 months to 12 years [53]. Three years after the diagnosis of HL, the childbirth rates of patients undergoing first-line CT only were found to be similar to the general population. No relationship between pregnancies and stage or treatment was found except for patients who relapsed or had refractory disease and underwent salvage CT with hematopoietic stem cell transplantation [54,55,57]. The authors could not find a higher reported prevalence of miscarriages, stillbirths, or birth defects compared to the healthy general population. These studies, however, were all subject to selection bias, being retrospective, and based on questionnaires or self-reported methods [54,55,56,57,58].

## 5. Conclusions

The risk of infertility after anticancer treatment must be discussed with male and female HL and DLBCL patients at the time of diagnosis. Semen cryopreservation should be offered to all patients before starting first-line treatment despite the low reported rate of utilization. This is because it is a simple, easy to perform, and relatively inexpensive procedure. Females over 35 years of age can be offered mature oocyte or ovarian tissue cryopreservation before first-line treatment containing alkylating agents. These options should not be routinely recommended to patients younger than 35 years who are receiving ABVD or R-CHO(E)P or R-EPOCH-DA (rituximab-etoposide, vincristine, doxorubicin, cyclophosphamide, vincristine-dose adjusted) regimens, but complete information about the pros and cons should be mandatory, and the decision of whether to adopt these fertility preservation procedures should follow the patients’ desires. For patients shifting to high cumulative dose alkylating-containing regimens, such as eBEACOPP, for positron emission tomography (PET) positivity after the first two ABVD cycles as well as for patients requiring salvage CT and ASCT, fertility preservation can be performed just before second-line treatment starts. GnRH analogs are not an alternative option to fertility preservation, but their administration during the period of treatment, starting at least 7 days before the administration of the first CT and continuing monthly during the whole period of CT, improves the rate of menstrual cycle recovery and reduces the risk of vaginal bleeding. With regard to the interval between the end of anticlastic treatment and conception, it seems more prudent to wait until the period of greatest risk of disease recurrence has passed. This period corresponds for both HL and DLBCL to the first two to three years after the end of first-line treatment for female patients.

Further research into the field of fertility preservation in lymphoma patients is still needed to better define POF, the optimal timing of gonadal function assessment, and ovarian function biomarkers. The following projects, recently adopted by the FIL, are part of international clinical research efforts in this topic: a survey about fertility preservation procedures among Italian hematology centers overseen by FIL, the results of which will be analyzed shortly; a feasibility study of the procedure of cryopreservation of mature oocytes and its long-term results in HL and DLBCL patients; and, finally, a retrospective study to assess long-term male fertility, which is ongoing.

## Figures and Tables

**Table 1:** Clinical questions and corresponding PICOs addressed by this systematic review.

***PICO 1***	
**1a.** Which female HL or DLBC adult premenopausal patients are eligible for fertility preservation procedures?	**P:** HL or DLBCL female patients ≥18 years before chemotherapy start; **I:** Chemotherapy regimens containing low cumulative doses of alkylating agents (e.g., ABVD) or high cumulative doses of alkylating agents (eBEACOPP, sBEACOPP, COPP, R-CHOP, R-da-EPOCH) and/or high-dose chemotherapy with autologous stem cell transplantation (ASCT) and/or pelvic radiotherapy; **C:** Healthy people; **O:** Incidence of amenorrhea and infertility in HL or DLBCL adult patients.
**1b.** Which male HL or DLBC adult patients are eligible for fertility preservation procedures?	**P:** HL or DLBCL male adult patients aged ≥18 years before chemotherapy start; **I:** Chemotherapy regimens containing low cumulative doses of alkylating agents (e.g., ABVD) or high cumulative doses of alkylating agents (eBEACOPP, sBEACOPP, COPP, R-CHOP, R-da-EPOCH) and/or high-dose chemotherapy with autologous stem cell transplantation (ASCT) and/or pelvic radiotherapy; **C:** Healthy people; **O:** Incidence of azoospermia and infertility in HL or DLBCL adult patients.
***PICO 2***	
**2a.** What are the fertility preservation procedures available to HL or DLBCL adult male patients?	**P2a:** HL or DLBCL adult male patients, aged ≥18 years, treated with chemotherapy regimens containing low cumulative doses of alkylating agents (e.g., ABVD) or high cumulative doses of alkylating agents (eBEACOPP, sBEACOPP, COPP, R-CHOP, R-da-EPOCH) or treated with high-dose chemotherapy (HD-CT) and autologous stem cell transplantation (ASCT) and/or radiotherapy (RT) on pelvic nodes; **I2a:** cryopreservation of spermatozoa collected through ejaculation, micro-TESE, testicular biopsy; **C2a:** none; **O2a:** incidence of azoospermia at least 12 months from end of treatment, hormone evaluation of testicular function (FSH, testosterone, inhibin serum levels), percentage of utilization of cryopreserved sperm, number of spontaneous conceptions and after assisted reproduction technique (ART) conceptions, number of live births.
**2b.** What are the fertility preservation procedures available to HL or DLBCL adult female premenopausal patients?	**P2b:** HL or NHL adult premenopausal female patients, aged ≥18 years, treated with chemotherapy regimens containing low cumulative doses of alkylating agents (e.g., ABVD) or high cumulative doses of alkylating agents (eBEACOPP, sBEACOPP, COPP, R-CHOP, R-da-EPOCH) or who had undergone HD-CT and ASCT and/or RT on inguino-iliac lymphnodes; **I2b:** cryopreservation of mature oocytes, cryopreservation of ovarian tissue, cryopreservation of immature oocytes; **C2b:** no therapy, estroprogestinic contraceptives, placebo; **O2b:** incidence of amenorrhea, regular menstrual cycle recovery, hormone evaluation of ovarian function (FSH, estradiol, AMH serum levels), spontaneous pregnancies, ART pregnancies, percentage of utilization of cryopreserved oocytes or ovarian tissue, number of live births.
***PICO 3***	
**3.** Is the use of gonadotropin-releasing hormone analogs (GnRHa) during treatment effective in reducing the risk of infertility in adult premenopausal female patients with HL or NHL DLBC, receiving CT regimens, HD-CT, or RT to pelvic nodes?	**P:** HL or DLBCL adult premenopausal female patients, aged ≥18 years, treated with chemotherapy regimens containing low cumulative doses of alkylating agents (e.g., ABVD) or high cumulative doses of alkylating agents (eBEACOPP, sBEACOPP, COPP, R-CHOP, R-da-EPOCH) or treated with HD-CT and ASCT and/or RT on inguino-iliac lymph nodes; **I:** GnRH agonists or antagonists in combination with chemotherapy and/or radiotherapy on the pelvic nodes; **C:** placebo, oral contraceptive pills, no therapy; **O:** maintenance or recovery of menstruation (normal or oligo-amenorrhea), hormonal evaluation of ovarian function (FSH, estradiol, AMH serum levels), spontaneous pregnancies after ≥12 months from the end of anticancer treatment
***PICO 4***	
**4a.** What are the most appropriate fertility investigations in adult male patients with HL or DLBC treated with CT and/or pelvic (inguino-iliac) irradiation?	**P:** HL or DLBCL adult male patients, aged ≥18 years, treated with chemotherapy regimens containing low cumulative doses of alkylating agents (e.g., ABVD) or high cumulative doses of alkylating agents (eBEACOPP, sBEACOPP, COPP, R-CHOP, R-da-EPOCH) or treated with high-dose chemotherapy (HD-CT) and ASCT and/or radiotherapy (RT) on inguino-iliac lymph nodes; **I:** Evaluation of gonadal reserve with sperm count; **C:** Evaluation of gonadal reserve with the measurement of blood hormone levels (FSH, LH, testosterone); **O:** incidence of infertility based on sperm count versus hormone level results, number of conceptions.
**4b.** What are the most appropriate fertility investigations in adult female patients with HL or DLBC treated with CT and/or pelvic (inguino-iliac) irradiation?	**P4:** HL or DLBC adult premenopausal female patients, aged ≥18 years, treated with chemotherapy regimens containing low cumulative doses of alkylating agents (e.g., ABVD) or high cumulative doses of alkylating agents (eBEACOPP, sBEACOPP, COPP, R-CHOP, R-da-EPOCH) or treated with high-dose chemotherapy (HD-CT) and ASCT) and/or radiotherapy (RT) on inguino-iliac lymph nodes; **I:** Evaluation of gonadal reserve with ultrasound (US) antral follicle count (AFC) measurement of serum AMH, FSH, estradiol level; **C:** Evaluation of gonadal reserve with menstrual pattern (history of menstrual cycle); **O:** incidence of infertility based on US and hormone levels versus menstrual history, number of conceptions.
***PICO 5***	
**5.** What is the best interval to respect from the end of HL or DLBCL treatment before conceiving?	**P:** HL or DLBCL adult males and females patients, aged ≥18 years, who have completed their anticancer treatment, willing to attempt parenthood; **I:** Less of 24 months or ≥24 months from the end of chemotherapy regimens containing low cumulative doses of alkylating agents (e.g., ABVD) or high cumulative doses of alkylating agents (eBEACOPP, sBEACOPP, COPP, R-CHOP, R-da-EPOCH) or treated with HD-CT and ASCT and/or RT on inguino-iliac lymph nodes; **C:** Healthy population comparable in age and sex; **O:** Number of spontaneous pregnancies, number of live births, number of miscarriages, number of stillbirths, number of congenital abnormalities.

## Supplementary Materials

The following are available online at https://www.mdpi.com/article/10.3390/cancers13122881/s1. Table S1: Search strategies. Table S2: Full-text eligibility and data extraction. Table S3: Risk of bias.
