# Supplementary material for: Male and Female Fertility: Prevention and Monitoring Hodgkin’ Lymphoma and Diffuse Large B-Cell Lymphoma Adult Survivors. A Systematic Review by the Fondazione Italiana Linfomi
Source: Cancers (Basel). 2021 Jun 9;13(12):2881. doi: 10.3390/cancers13122881 (PMC8228520; doi:10.3390/cancers13122881)
Supplement: Supplementary file 1 [file cancers-13-02881-s001.zip › cancers-Table S1.pdf]

cancers

MDPI

Systematic Review

Male and Female Fertility: Prevention and Monitoring Hodgkin' Lymphoma and Diffuse Large B-Cell Lymphoma Adult Survivors. A Systematic Review by the Fondazione Italiana Linfomi

Simonetta Viviani [1,*], Valentina Caccavari [2], Chiara Gerardi [3], Safaa Ramadan [1,4], Eleonora Allocati [3], Carla Minoia [5], Attilio Guarini [5] and Anna Di Russo [6]

1 Division of Onco-Hematology IEO, European Institute of Oncology IRCCS, 20141 Milan Italy; safaa.ramadan@ieo.it
2 Assisted Reproduction Unit, Istituto Clinico Città Studi, 20131 Milan, Italy; valentina.caccavari@ic-cittastudi.it
3 Istituto di Ricerche Farmacologiche "Mario Negri" IRCCS, 20156 Milan, Italy; chiara.gerardi@marionegri.it (C.G.); eleonora.allocati@marionegri.it (E.A.)
4 Medical Oncology Dep., National Cancer Institute, Cairo University, 11796 Cairo, Egypt
5 Hematology Unit—IRCCS Istituto Tumori "Giovanni Paolo II", 70124 Bari, Italy; carlaminoia@libero.it (C.M.); attilioguarini@oncologico.bari.it (A.G.)
6 Department of Radiation Oncology, Fondazione IRCCS Istituto Nazionale dei Tumori di Milano, 20133 Milan, Italy; anna.dirusso@istitutotumori.mi.it
* Correspondence: simonetta.viviani@ieo.it

Citation: Viviani, S.; Caccavari, V.; Gerardi, C.; Ramadan, S.; Allocati, E.; Minoia, C.; Guarini, A.; Di Russo, A. Male and Female Fertility: Prevention and Monitoring in Hodgkin Lymphoma and Diffuse Large B-Cell Lymphoma Adult Survivors. A Systematic Review by the Fondazione Italiana Linfomi. *Cancers* **2021**, 13, 2881. https://doi.org/10.3390/cancers13122881

Academic Editor: Francesco Merli, Monica Balzarotti and Alessandra Tucci

Received: 1 May 2021
Accepted: 5 June 2021
Published: 9 June 2021

Publisher's Note: MDPI stays neutral with regard to jurisdictional claims in published maps and institutional affiliations.

Copyright: © 2021 by the authors. Licensee MDPI, Basel, Switzerland. This article is an open access article distributed under the terms and conditions of the Creative Commons Attribution (CC BY) license (http://creativecommons.org/licenses/by/4.0/).

Table S1. Example of search strategy used in MedLine and adapted to search the other databases.

• PICO 1: eligibility to fertility preservation procedures for female and male with HL or DLBC<br>• MedLine, Embase, and Cochrane databases up to September 2020<br>((((((((("Fertility Preservation"[Mesh]) OR fertility preservation) OR "Cryopreservation"[Mesh] OR cryopres*))))) AND ((((((hodgkin disease[Title/Abstract]) OR "Hodgkin Disease"[Mesh]) OR hodgkin lymphoma) OR "Lymphoma, Large B-Cell, Diffuse"[Mesh]) OR diffuse large B cell lymphoma))))
• PICO 2: male cryopreservation procedures<br>MedLine, Embase, and Cochrane databases up to September 2020<br>(((((((hodgkin* lymphoma[Title/Abstract]) OR hodgkin* disease[Title/Abstract])) OR ((Lymphoma, Large B-Cell, Diffuse[MeSH Terms]) OR Hodgkin Disease[MeSH Terms])) OR diffuse large b-cell lymphoma[Title/Abstract])) AND (((("Semen Preservation"[Mesh]) OR "Cryopreservation"[Mesh])) OR (((((semen preserv*[Title/Abstract]) OR semen cryo*[Title/Abstract]) OR sperm preserv*[Title/Abstract]) OR sperm cryo*[Title/Abstract]) OR male fertil*)))
• PICO 2: female cryopreservation procedures<br>MedLine, Embase, and Cochrane databases up to September 2020<br>(((hodgkin* lymphoma[Title/Abstract] OR hodgkin* disease[Title/Abstract] OR Lymphoma, Large B-Cell, Diffuse[MeSH Terms] OR Hodgkin Disease[MeSH Terms] OR diffuse large b-cell lymphoma[Title/Abstract])) AND (((((((("Fertility Preservation"[Mesh]) OR "Oocyte Retrieval"[Mesh]) OR "Cryopreservation"[Mesh]) AND Humans[Mesh] AND Female[MeSH Terms])) OR (((oocyte* preserv*[Title/Abstract] OR oocyte* cryo*[Title/Abstract] OR female fertil*[Title/Abstract])) AND Humans[Mesh])))
• PICO 3: sterility risk reduction and use of of gonadotropin-releasing hormone analogues (GnRHa)<br>MedLine, Embase, and Cochrane databases up to September 2020<br>(((((((((((""gonadorelin"") OR ""leuprorelin"") OR ""triptorelin"") OR ""enantone"") OR ""gnrh antagonist"") OR ""gnrh agonist"") OR ""decapeptyl"") OR ""buserelin"") OR ""goserelin"") OR ""gnrh analog"") OR GnRH analog)) AND (((""Hodgkin Disease""[Mesh](hodgkin disease) OR ""Hodgkin Disease""[Mesh] OR (hodgkin lymphoma) OR (diffuse large b cell lymphoma) OR ""Lymphoma, Large B-Cell, Diffuse""[Mesh])) AND FEMALE)
• PICO 4 : appropriate fertility investigations in male<br>MedLine, Embase, and Cochrane databases up to September 2020<br>((((((("Lymphoma, Non-Hodgkin"[Mesh]) OR "Lymphoma, Non-Hodgkin"[Majr])) OR (("Hodgkin Disease"[Mesh]) OR "Hodgkin Disease"[Majr]))) AND (((("Fertility"[Mesh]) OR "Fertility"[Majr])) OR (("Male"[Mesh]) OR "Male"[Majr])))) AND ((((("Follicle Stimulating Hormone, Human"[Mesh]) OR "Follicle Stimulating Hormone, Human"[Majr])) OR (("Luteinizing Hormone"[Mesh]) OR "Luteinizing Hormone"[Majr])) OR (("Testosterone"[Mesh]) OR "Testosterone"[Majr])) Sort by: PublicationDate
• PICO 4:appropriate fertility investigations in female<br>MedLine, Embase, and Cochrane databases up to September 2020<br>(((((((("Lymphoma, Non-Hodgkin"[Mesh]) OR "Lymphoma, Non-Hodgkin"[Majr])) OR (("Hodgkin Disease"[Mesh]) OR "Hodgkin Disease"[Majr]))) AND (((("Female"[Mesh]) OR "Female"[Majr])) OR (("Fertility"[Mesh]) OR "Fertility"[Majr]))) AND ((((("Anti-Mullerian Hormone"[Mesh]) OR "Anti-Mullerian Hormone"[Majr])) OR (("Luteinizing Hormone"[Mesh]) OR "Luteinizing Hormone"[Majr])) OR (("Follicle Stimulating Hormone, Human"[Mesh]) OR "Follicle Stimulating Hormone, Human"[Majr])))) AND (((((("Groin"[Mesh]) OR "Pelvis"[Mesh]) OR "Radiotherapy"[Mesh]) OR "Radiotherapy"[Majr])) OR (("Drug Therapy"[Mesh]) OR "Drug Therapy"[Majr])) Sort by: PublicationDate
• PICO 5:– time to conception<br>MedLine, Embase, and Cochrane databases up to September 2020<br>((((((("Lymphoma, Non-Hodgkin"[Mesh]) OR "Lymphoma, Non-Hodgkin"[Majr])) AND (("Hodgkin Disease"[Mesh]) OR "Hodgkin Disease"[Majr]))) AND ("reproductive physiological phenomena"[MeSH Terms] OR ("reproductive"[All Fields] AND "physiological"[All Fields] AND "phenomena"[All Fields]) OR "reproductive physiological phenomena"[All Fields]))) AND (("Pregnancy"[Mesh]) OR "Pregnancy"[Majr]) Sort by: PublicationDate
